# Identifying Psychosocial, Self-Management, and Health Profiles Among Women With Chronic Pain Who Have Experienced Intimate Partner Violence and Those Who Have Not: Protocol for a 2-Phase Qualitative and Cross-Sectional Study Using AI Techniques

**DOI:** 10.2196/66396

**Published:** 2025-07-08

**Authors:** Ainara Nardi-Rodríguez, Sónia Bernardes, María Ángeles Pastor-Mira, Sofía López-Roig, Lidia Pamies-Aubalat, Andrés Sánchez-Prada, Victoria A Ferrer-Pérez, Ignacio Rodríguez-Rodríguez, Purificación Heras-González

**Affiliations:** 1 Universidad Miguel Hernández Sant Joan D´Alacant Spain; 2 ISCTE-Instituto Universitário de Lisboa Lisboa Portugal; 3 Universidad Miguel Hernández Elche Spain; 4 Pontifical University of Salamanca Salamanca Spain; 5 Universitat de les Illes Balears Palma Spain; 6 Universidad de Málaga Málaga Spain

**Keywords:** chronic pain, intimate partner violence against women, treatment, risk factors, profiles, qualitative study, quantitative study, artificial intelligence, AI

## Abstract

**Background:**

Women who experience intimate partner violence (IPV) are more likely to develop disabling chronic pain (CP). However, there is little information on what it means to live with CP while being exposed to IPV. In addition, despite well-established risk and protective factors for CP and its health outcomes, there are no data on whether these factors differ in women who have experienced IPV compared to those who have not.

**Objective:**

Our aims are to understand the meaning and implications of living with CP for women who have experienced IPV compared to women with CP alone and to identify possible differences in risk and protective factors as well as health outcomes.

**Methods:**

We have designed 2 studies to be conducted in 2 phases. The first phase will involve a qualitative study with a descriptive and exploratory design. Individual semistructured interviews will be conducted with at least 10 women with CP alone and with 10 women with CP who have experienced IPV. Reflexive thematic analysis will be used to examine participants’ experiences, meanings, and realities. In the second phase, a cross-sectional study will be conducted with women with CP affected by IPV, including those currently experiencing IPV and those with past exposure to IPV, as well as women with CP who have never experienced IPV. A total of 359 women will complete various scales assessing risk and protective factors (eg, pain-related worrying and activity patterns) and health outcomes (eg, legal and illegal drug use and health care use). Data analysis will involve inferential statistics and machine learning.

**Results:**

As of May 2025, we had contacted relevant associations to present the project and begin recruiting voluntary participants. We have conducted the pilot study to assure the adequacy of the questionnaire and usability or technical functionality of the web-based platform. The results are expected to be published starting in January 2026.

**Conclusions:**

This research addresses two urgent needs in pain research identified by the European Pain Federation: (1) expanding knowledge on CP management in relation to contextual factors; and (2) identifying specific psychosocial, self-management, and health profiles among women with CP who have experienced IPV and those who have not. This could provide valuable insights for personalizing pain management treatments. In addition, the findings may help identify women experiencing IPV who present to health care settings.

**International Registered Report Identifier (IRRID):**

PRR1-10.2196/66396

## Introduction

### Background

Chronic pain (CP) is defined as pain that persists beyond the normal tissue healing period, typically lasting approximately 3 months. It is a major health concern affecting approximately 30% of people worldwide [1]. CP is more prevalent and burdensome among populations experiencing vulnerability, including women, older adults, children, individuals from racial or ethnic minority groups, and those from lower socioeconomic backgrounds [2,3]. In Europe, the economic burden of CP could reach up to €300 billion annually (US $343 billion) [4].

There is broad consensus that understanding and treating CP requires a biopsychosocial approach. Beyond biological and physiological factors, psychological, social, and life experiences play a crucial role in the development, exacerbation, and persistence of pain [5-7]. Notably, CP is a well-documented consequence of another public health problem, intimate partner violence (IPV) against women, as demonstrated by controlled studies in both population-based and clinical samples [8-11].

IPV against women is a gender-based issue [12-14] that encompasses physical, psychological, or sexual violence perpetrated by some men against their female partners [15]. Today, IPV against women remains a widespread epidemic, affecting at least 30% of women worldwide [16]. It has severe health and social consequences [17] and imposes an economic cost of €174 billion (US $199 billion) per year on the European Union [18].

Compared to women who have not experienced violence, those exposed to IPV against women report a higher prevalence of fibromyalgia, abdominal pain, genitourinary tract pain, pelvic and genital pain, headaches (including migraines), and back and neck pain [19-22]. A study conducted with 108 male and female patients with CP attending a rehabilitation center found that 56% had a current or past history of IPV, with more than 80% being women. Nearly 30% had experienced IPV within the previous year [23].

Other studies have shown that those who have experienced IPV and are living with sickle cell disease are 5 times more likely to report CP than those who have never experienced IPV [24]. In addition, they are nearly twice as likely to develop fibromyalgia and chronic fatigue syndrome [25]. A recent systematic review and meta-analysis found strong and consistent associations between women who have experienced violence—whether by a partner or others—and CP conditions [26]. Furthermore, IPV against women is often linked to disabling pain [8,23,27]. Survivors with disabilities are more likely to report generalized CP than those with other disabling health conditions [28], and more than one-third continue to experience a high level of pain-related disability long after ending the abusive relationship [29].

Women who have experienced IPV also frequently use medication to alleviate pain [30]; for example, individuals with sickle cell disease who have experienced interpersonal violence are more than 6 times as likely to use opiate-based medications daily compared to those without a history of violence exposure [24].

Given the increased likelihood of those with exposure to IPV against women developing disabling CP and experiencing worse health outcomes [23,24,28,30]—even after leaving a violent relationship [29]—it is possible that experiencing (or having experienced) IPV against women influences the risk and protective factors involved in the development and maintenance of disabling CP differently. While previous studies have examined factors that predict or contribute to CP in those with exposure to violence [31,32], to our knowledge, no comparative studies have explored whether the underlying factors of CP differ based on a history of interpersonal violence.

Gathering this information is crucial for early detection and tailored treatment strategies for CP management in women who have experienced IPV as well as those who have not. Some researchers suggest that women with CP who are experiencing IPV may require more holistic care and support [26]. In addition, there is a need to better understand the daily experience of women living with CP while currently experiencing IPV compared to that of women who experience CP without a history of experiencing violence. However, no studies on this topic were found in the reviewed literature. In fact, the intersection of CP and IPV against women as interrelated phenomena has received little research attention [23].

### Goals of This Study

This research project has 2 general objectives and 6 specific aims.

First, we seek to understand the meaning and implications of living with CP for women experiencing IPV compared to those with CP alone. From this point forward, the term “women with CP alone” will refer to women who have never experienced any form of psychological, physical, or sexual violence at any stage of their lives, whether perpetrated by an intimate partner or another known or unknown individual. We include other forms of violence beyond IPV against women because they may also influence CP management and health outcomes [31].

More specifically, by comparing women with CP and exposure to IPV to women with CP alone, we aim to (1) examine how CP affects their daily lives, (2) identify the impact that CP may have on their intimate relationship and vice versa, (3) understand how they self-manage CP, and (4) describe their experiences with health systems.

Second, we aim to determine whether women with CP alone, women with CP who are currently experiencing IPV, and women with CP who have a past history of IPV present distinct profiles based on risk and protective factors for disabling CP and health outcomes. If differences exist, we will also (5) identify the factors that best explain these profiles and (6) detect more specific subprofiles within each group.

Ultimately, the findings will be used to develop assessment and intervention guidelines for health and social service professionals.

### Risk and Protective Factors

#### Overview

The scientific literature has identified well-known risk and protective factors for disabling CP. The most widely studied have been individual factors [5,33] that are “the result of historical and ongoing interactions between genetic, epigenetic, and environmental conditions, which are embedded in and shaped by multilevel social factors” [34].

However, the role of interpersonal factors—such as experiencing IPV against women versus not experiencing it—in shaping these “individual” factors is often overlooked, despite evidence recognizing interpersonal relationships as both a risk and a protective factor for pain-related dysfunction [33,35]. In the following subsections, we provide a brief overview of some of the most relevant risk and protective factors for CP and health outcomes identified in the scientific literature.

#### Gender Norms and Identity

Biological differences partially explain sex-related variations in pain perception; however, psychosocial factors such as gender norms also play a significant role. Gender norms influence differences between men and women in pain sensitivity, pain tolerance, pain expression, and coping mechanisms [36-39]. These norms shape gender identity, which refers to an individual’s social and psychological self-conception as a man or a woman [40].

A recent clinical study pointed out that gender identity, rather than genetic sex, may play a more significant role in shaping pain perception [41]. Clinical studies have found that feminine traits correlate with pain sensitivity and can predict the development of CP in men [42,43]. In addition, traits associated with femininity, such as servility or subordination, have been linked to greater disability in individuals with CP [44]. More recently, a study based on machine learning modeling identified a strong association between agreeableness and fibromyalgia [6].

#### Education and Socioeconomic Background

Research highlights socioeconomic factors—such as income, occupation, wealth, and education—as primary contributors to a wide range of health outcomes [45], including perceived pain and CP management [5,46]. In fact, socioeconomic status is inversely related to both the prevalence and severity of CP, as well as higher levels of disability [47,48].

Regarding education, having spent less time in education can influence pain in various ways, including through exposure to environmental stressors, work history, and health literacy [46]. Education level has also been linked to the overall burden on health care systems [49].

#### Ethnicity or Racialized and Minoritized Groups

Through social learning, children acquire the specific cultural meanings attached to pain, which is influenced by ethnicity [50,51]. Ethnicity impacts various aspects of pain experiences, such as pain expression, remedies, when to ask for help, and perceptions of health care systems and illness behaviors [52]; for example, children learn when it is appropriate to share pain experiences with family members and caregivers [53,54].

Studies in the United States and Europe have shown differences across ethnic and cultural groups (eg, American, British, Latinx, and South Asian) in self-reported pain intensity and coping styles [55-60]. In addition, there is increasing evidence that racism negatively affects pain perception and pain management [61-63].

#### Pain Worrying (Pain Catastrophizing)

Pain catastrophizing, now referred to as pain worrying [64], is considered one of the strongest predictors and correlates to negative pain experiences [65-69]. The higher the levels of pain worrying, the greater the intensity, severity, and emotional distress related to pain [65,70,71]. Pain worrying is also associated with a greater need for medical advice and increased health care use [72], as well as higher levels of disability [73]. It is a key factor in explaining variability in pain experiences [67] and is commonly used as a criterion to assess changes in cognition, making it an important component in interventions aimed at reducing pain and improving functioning [74]. The fear-avoidance model, one of the most accepted theories on CP, suggests that pain worrying mediates the relationship between pain and disability [75].

#### Pain-Related Self-Efficacy

Self-efficacy refers to an individual’s belief in their ability to organize and execute the necessary actions to achieve specific goals in a given situation [76]. For individuals with CP, self-efficacy is related to their belief in their ability to perform activities despite pain—referred to as pain-related self-efficacy [77]. Regardless of pain severity, improvements in pain-related self-efficacy are associated with reductions in disability [78]. Moreover, a previous meta-analysis found that pain-related self-efficacy negatively correlated with impairment, affective distress, and pain severity [79].

#### Traumatic Events

The scientific literature has strongly linked posttraumatic stress disorder (PTSD), particularly as a result of childhood trauma, to CP [23,80]. The comorbidity between PTSD and CP health issues ranges from 10% to 50% [81]. According to some studies, when CP coexists with PTSD, the pain tends to be more severe and causes greater distress, higher levels of interference, and more disability compared to cases of CP without a history of trauma [82]. PTSD is also a highly prevalent consequence of IPV against women, affecting 31% to 84.4% of women experiencing IPV [83], with similar rates found in more recent studies [84].

#### Receipt of Informal Social Support

Social support refers to the resources provided through both informal and formal relationships [85]. There is growing research on how social factors, particularly social support, influence pain experiences [86-88]. Informal social support is especially valued by patients with CP [89,90]. Within a person’s social context, spouses or partners can play a critical role in CP management as either risk or protective factors. They can promote autonomy by encouraging positive behaviors [91] or foster dependence by reinforcing disabling pain behaviors through overprotectiveness [91-93] or negative responses such as hostility and criticism [93,94]. However, negative responses are more likely to worsen pain intensity, disability, and pain behaviors when they occur in the context of low marital satisfaction [95,96].

#### Activity Patterns

Studies have identified 3 general activity patterns that affect the quality of life and CP adjustment: pacing, avoidance, and persistence [97]. These general activity patterns can be divided into more specific ones depending on the person’s goals [98,99]. However, to simplify, pacing generally involves breaking down daily activities into smaller, more manageable tasks and distributing them throughout the day. Pacing is used in clinical contexts to reduce avoidance and excessive persistence. There is strong empirical evidence that avoidance and excessive persistence contribute to worsened CP outcomes, including poorer psychological and physical functioning, increased pain [97,100], and difficulties in performing daily activities, leading to more global disability [101,102]. Avoidance is a strategy aimed at minimizing pain escalation by reducing engagement in physical activities. In cases of highly disabling CP, avoiding physical activity is common [103]. Conversely, the persistence pattern involves continuing to perform tasks despite pain, aiming to increase productivity by “ignoring” the pain [97].

#### Exercising

Exercising is a key component of the multidisciplinary treatments in CP, offering significant benefits beyond musculoskeletal health. It has been shown to reduce pain intensity and fatigue as well as improve physical function, psychological well-being, and overall quality of life [104,105]. Its benefits for CP go beyond just musculoskeletal health [106,107]. In fact, several studies have shown that exercise ameliorates pain even if the person’s strength, flexibility, or endurance does not improve [107,108]. It could be that physical exercise has analgesic effects [109].

#### CP Outcomes

How individuals manage CP can have deep and long-lasting effects on their quality of life. Effective management can minimize pain, reduce physician visits, empower patients to participate in treatment decisions, and provide a sense of control over their lives [110-114]. By contrast, poor CP management can negatively impact socialization, reduce participation in social events, and decrease independence, leading to isolation [115,116]. It can also increase vulnerability to suicide and suicidal ideation [110] as well as contribute to the misuse of both licit and illicit drugs—such as alcohol, marijuana, or heroin—for pain relief [117-119], which, in turn, further strains health systems. Pain is one of the primary reasons for medical consultations worldwide, accounting for 22% of visits [120,121]. People with CP visit their general practitioner twice as often as those without CP [122], and they are more likely to use emergency services and unscheduled care [123,124]. In Spain, CP is the second most common reason for primary care consultations, with >50% of visits related to this condition [125].

## Methods

This research project is expected to last 3 years and will be conducted in 2 phases: the first phase will involve a qualitative study (general aim 1, study 1), and the second phase will involve a quantitative cross-sectional study (general aim 2, study 2). Before commencing the studies, the web-based closed questionnaires to be completed by participants will be piloted with a sample of 10 women to address any issues related to content, usability, or technical functionality of the web-based platform used to design the questionnaires.

### Ethical Considerations

Approval has been obtained from the ethics committee of Miguel Hernández University (DCC.ANR.240227) and the Public Health Ethics Committee for Research of the Foundation for the Promotion of Health and Biomedical Research of the Valencian Community (CEI-SP 20240531/05/P).

Ethical requirements will be consistent across both studies. All participants will receive a letter with a description of the study and of the individual interview content and questionnaires that they will have to fill in depending on the study in which they participate. There will not be any incentive for participation.

For the qualitative phase (phase 1), participants will sign an informed consent form, which clearly states that participation is voluntary and that they can withdraw at any time. Before the interviews, they will have to complete a web-based questionnaire to check whether they meet the inclusion criteria. Participants will submit their informed consent to a designated professional at the relevant health center, unit, or association, who will assign a code to each participant and will also be responsible for ensuring the correct completion of the web-based questionnaires with the corresponding code. Participants who do not meet the inclusion criteria will have their questionnaires deleted, and the informed consent forms for nonparticipants will be discarded by the professionals. Participants who meet the inclusion criteria will be interviewed. Interviews will be audio recorded for analysis purposes and erased afterward. The informed consent forms will be securely handed over by the professionals to the principal investigator in a sealed envelope. The professionals will also schedule the interview date and communicate the participant’s code number to the research team, ensuring that the interview and questionnaire data match. The research team will not have access to any personal identifying information, such as names.

For the quantitative phase (phase 2), participants will fill in anonymous web-based questionnaires, as outlined in the informed consent form. For security reasons, participants must complete the questionnaire at the center they attend. Allowing them to complete it at home could put them at risk because their aggressors could see their responses. Participants will submit their informed consent to a designated professional at the relevant health center, unit, or association. This professional will have a list of codes. They will have to assign one to each participant (unknown to them) and will have to enter it so that each participant can start or pick up the questionnaire where they left off the last time. The professional will collect all informed consent forms and submit them to the principal investigator in a sealed envelope. Throughout the study, the research team will not have access to any personal identifying information.

In both studies, the web-based closed questionnaires will be designed using LimeSurvey, a private platform that meets security standards and data protection regulations. The responses will be stored in a database under the supervision of one of the collaborating universities. Before commencing the studies, the questionnaires will be piloted with a sample of 10 women to address any issues related to content, usability, or technical functionality of the web-based platform.

### Phase 1: Qualitative Study (General Aim 1, Specific Objectives 1 to 4)

#### Overview

We will use a qualitative approach with a descriptive and exploratory design, conducting individual semistructured interviews. Focus groups are not considered due to the necessity of maintaining the anonymity of those experiencing IPV against women. The results of this phase will allow us to incorporate any additional relevant factors or health outcomes into the study’s second phase.

#### Participants

The study will be conducted in the province of Alicante in Spain. To detect possible differences, interviews will be conducted with 2 groups: first, adult women with CP who are currently experiencing IPV (group 1) and, second, women with CP who are in a partner relationship and have never experienced IPV or any other form of violence, as defined earlier (group 2).

We will initially interview 10 women from each group, progressively increasing the number of interviews until theme saturation is reached [126]. Both groups will include women with varying sociodemographic characteristics, levels of disabling pain, and trauma symptoms. However, the diversity between the 2 groups will be balanced, ensuring that both groups include equal numbers of women with disabling pain and those without.

#### Procedure

First, we will reach out to the organizations through which we plan to access both groups and request their collaboration. To contact women who have experienced or are experiencing IPV and women with CP, we will consult associations and support centers for IPV against women, CP associations, CP units, and primary health care centers. These organizations will be asked to inform their patients about the project and encourage their participation.

Eligible candidates will be required to complete a series of questionnaires (refer to the Instruments subsection) to determine whether they meet the inclusion and exclusion criteria. The data from those who do not meet the criteria will be discarded. Those who qualify will proceed to complete additional questionnaires. The answers from these questionnaires will help us form the 2 groups while ensuring diversity based on age, racial background, and degree of disabling CP.

The inclusion criteria for both groups will be as follows: (1) women aged ≥18 years, (2) in a heterosexual intimate relationship for at least 1 year (as per Spanish law, IPV against women pertains to violence perpetrated by male intimate partners) [15], and (3) living with a CP condition highly prevalent in women and among those who have experienced IPV against women (eg, fibromyalgia, abdominal pain, genitourinary tract pain, pelvic and genital pain, headaches [including migraines], back and neck pain, and chronic fatigue syndrome) [19-22]. Women belonging to group 1 will, in addition, have to be currently experiencing IPV.

The exclusion criterion for group 2 will be having previously experienced IPV against women or any other form of violence because this may affect their current intimate relationships. The exclusion criterion for both groups will be experiencing oncological pain.

#### Interviews

A member of the research team will be in charge of conducting individual semistructured interviews. For security reasons, the interviews will be carried out at the locations where participants were recruited. Each interview is expected to last approximately 1 hour. The questions that will be posed to both samples are as follows:

- What does “living with CP” mean to you?
- How do you manage CP in your daily life?
- Have your self-management strategies changed over time? If yes, how?
- What role does your partner play with regard to your CP?
- Does CP affect your relationship? If yes, how?
- Does your partner use your health condition against you? If yes, can you provide examples?
- Does your relationship affect your CP? If yes, how?
- What is your experience with the health system?
- What is your relationship with medication?

#### Instruments

The variables and instruments that will be used, organized by purpose, are summarized in Table 1 and described in the subsections that follow.

**Table 1:** Variables and instruments used in study 1, organized by purpose.

Variable	Questionnaire	Control purposes^a^	Data analysis
Chronic pain	Ad hoc question	✓	
Sociodemographic characteristics	Ad hoc questionnaire	✓	✓
IPV^b^ against women	European Union Agency for Fundamental Rights study on violence against women survey inventory	✓	✓
Possible traumatic events experienced	Adapted version of Life Events Checklist for DSM-5	✓	
Disabling chronic pain	GCPS-R^c^		✓
Severity of PTSD^d^ symptoms	EGS-R^e^		✓

^a^We include compliance with the inclusion and exclusion criteria.

^b^IPV: intimate partner violence.

^c^GCPS-R: Graded Chronic Pain Scale Revised.

^d^PTSD: posttraumatic stress disorder.

eEGS-R: Escala de Gravedad de Síntomas Revisada (Posttraumatic Stress Disorder Symptom Severity Scale-Revised [according to Diagnostic and Statistical Manual of Mental Disorders, Fifth Edition criteria]).

#### Screening for Compliance With Inclusion and Exclusion Criteria

### CP and Type

To distinguish participants with acute pain from those with CP, they will first be asked how long they have been experiencing pain. Participants will also select the type of CP they experience, such as fibromyalgia, from a provided list.

### Sociodemographic Characteristics

Participants will have to indicate their age, sex at birth, gender identification, racial and ethnic identity, sexual orientation, and relationship status (whether currently in a relationship and for how long).

### IPV Against Women: European Union Agency for Fundamental Rights Study on Violence Against Women Survey Inventory

Participants will complete an inventory [127] consisting of 32 items that assess various forms of violence exerted by a partner: 11 items on physical violence, 4 on sexual violence, and 17 on emotional and psychological violence. Participants will respond with the frequency of each type of violence (0=*never*, 1=*once*, and 2=*more than once*) and specify whether the violence was inflicted by a past or current partner, including the duration of the violent relationship. A woman will be classified as experiencing current IPV or having experienced past IPV if she reports experiencing any item more than once from the same partner.

### Possible Traumatic Events Experienced: Adapted Version of Life Events Checklist for DSM-5

The original version of this checklist [128] contains 17 items referring to different stressful events. Participants will have to mark whether they have experienced, witnessed, or learned about each event; whether it was part of their job; or whether it does not apply to them. The adapted version will rephrase the items on physical and sexual assault (items 6 to 9) into a single item: exposure to “psychological, physical, and/or sexual violence or abuse at any stage of your life by a known or unknown person other than an intimate partner.”

The purpose of using this instrument is helping to control for any external traumatic events (eg, natural disasters) that may influence pain experiences different from experiencing IPV.

#### Configuration for Diverse Groups and for Data Analysis Purposes

##### Additional Sociodemographic Characteristics

Both groups of participants will need to complete a questionnaire detailing their nationality, years of education, profession, annual household and personal incomes, relationship satisfaction, and the number of children. This information will help in differentiating the groups and understanding various factors that may impact pain experiences. Regarding pain characteristics, participants will report on pain location, severity, diagnosis, and the time since they were diagnosed with CP.

##### IPV Against Women: European Union Agency for Fundamental Rights Study on Violence Against Women Survey Inventory

This inventory [127] will be used to assess the severity of violence experienced by participants, based on the total score obtained, with a maximum possible score of 64 points. The severity of the violence will be classified into 3 categories—low, moderate, and high—determined by calculating percentiles. This inventory is available in multiple European languages, including Spanish.

##### Disabling CP: Graded CP Scale Revised

For assessing disabling CP, the Graded Chronic Pain Scale Revised [129] will be used. This scale consists of 5 items, each rated on a scale ranging from 0 to 10, and provides a simple and valid method for classifying CP as mild, bothersome, or high impact. Those who, on items 1 and 2, report that in the past 3 months, their pain has limited their life activities or work on most days or every day will be classified as grade 3 (high-impact CP). Those who score ≥12 on items 3 to 5 will be classified as grade 2 (bothersome CP); and those who score <12, with no high-impact pain, will be categorized as grade 1 (mild CP). The Graded Chronic Pain Scale Revised has demonstrated good concurrent validity.

##### Severity of PTSD Symptoms: PTSD Symptom Severity Scale-Revised

This instrument [130] consists of 21 items related to the *Diagnostic and Statistical Manual of Mental Disorders, Fifth Edition* diagnostic criteria: 5 to re-experiencing symptoms, 3 to behavioral and cognitive avoidance symptoms, 7 to cognitive disturbances and negative mood, and 6 to symptoms of increased psychophysiological arousal and reactivity. The responses are rated on a 3 points Likert- type scale ranging from 0 to 3 points according to symptom frequency and intensity. When a person scores at least 2 points in an item, the symptom to which the item refers to is considered present. The overall scale ranges from 0 to 63 points. The instrument has shown high internal consistency, good discriminant and concurrent validity and diagnostic efficacy in a large sample of Spanish women who have mostly experience IPV.

#### Data Analysis

The verbatim interview transcripts will undergo inductive reflexive thematic analysis, a foundational and flexible qualitative method that allows researchers to identify, analyze, and report themes or patterns in data [131,132]. In this study, the focus will be on understanding participants’ experiences, meanings, and realities.

Two researchers trained in reflexive thematic analysis will collaborate using the process established by Braun and Clarke [131,132]. The process begins with data familiarization, where the researchers review the material and take initial notes. Next, they will systematically code the data, group similar codes, and produce initial themes based on the collated data. These themes will then be developed and revised through an iterative process. The final stage involves refining, defining, and naming the themes before writing the report. Inductive coding will be carried out using NVivo 10 software (Lumivero).

To ensure the quality of the analysis, several procedures will be implemented throughout the research. This will include ensuring that the research question aligns with the methodological decisions (ie, methodological coherence) and selecting participants who can provide the most relevant and insightful information. Data analysis will be an iterative process, allowing ideas to evolve and be confirmed with the data. In addition, the research team will work closely together to develop interpretations and concepts that best capture the phenomenon under investigation.

#### Phase 2: Quantitative Study (General Aim 2, Specific Objectives 5 and 6)

Phase 2 of the research will focus on a cross-sectional quantitative study aimed at examining the differences between 3 groups of women.

#### Participants

The reference population will include heterosexual women aged ≥18 years living in the province of Alicante. To detect possible differences in profiles, the study will be carried out in 3 groups of women: those who have CP and have never experienced IPV or any other type of violence (group 1), those who have CP and are currently experiencing IPV (group 2), or those who have CP and have a past history of IPV (group 3). It is important to note that the effects of violence may continue to impact individuals long after the relationship ends, and this study aims to identify risk and protective factors, as well as health outcomes, to support detection and intervention efforts.

#### Sample Size Calculation

To calculate the sample size, the prevalence of the pain-avoidance activity pattern was used, which is typically present in cases of highly disabling CP [102]. Specifically, we considered several factors: first, the expected prevalence ratio assumed to be 2.0 between individuals with CP who are exposed to IPV against women and those who are not. Second, we estimated the prevalence of the pain-avoidance activity pattern within the study population, assuming it to be approximately 20%. While a previous study found that 40.4% of patients with fibromyalgia exhibited this pattern [133], we decided to use a more conservative estimate, given that the prevalence of this behavior is likely lower among the broader population of patients with CP. Finally, we accounted for the expected prevalence of IPV exposure among women with CP, estimated at 30% based on findings from earlier research [23].

We have defined the risk level α as 5% and the study power as 90%. The program used for size calculation was a macro, !NSize, for SPSS Statistics [134]. On the basis of these parameters, the required sample size consists of 359 women with CP: 193 (53.8%) with CP alone, 83 (23.1%) with CP and a current history of IPV, and 83 (23.1%) with CP and a past history of IPV.

#### Procedure

We will access the sample through the same channels as in phase 1. First, we will explain the benefits of the project to the organizations and request their support in encouraging their patients to participate. The directors of these organizations will receive the link to the web-based questionnaire, which will then be distributed to patients who, for security reasons, can only complete the questionnaire at the respective facilities.

Participants willing to participate will have to complete a web-based questionnaire. The first page will outline the objectives of the study, and the second page will ask participants to confirm whether they meet the inclusion criteria, as described in the phase 1 Procedure subsection. Specifically, participants must be women aged ≥18 years and must have been living with one of the identified types of CP for at least 3 months.

Participants will have 1 month to complete the questionnaire. During this period, answers will be saved in the system to allow participants to complete the questionnaire on different occasions; however, as stated, the professionals at the centers will need to activate the survey links. Survey links are deactivated to prevent respondents from completing the questionnaires outside of the designated locations. The data will be automatically stored in a database, and the progress of sample collection will be monitored weekly to ensure that each group reaches the necessary sample size.

#### Variables and Instruments

The variables and instruments that will be used, organized by purpose, are summarized in Table 2 and described in the subsections that follow. Some response scales will be alternated to identify biases in participants’ responses.

**Table 2:** Variables and instruments used in study 2, organized by purpose.

Variable	Questionnaire	Control purposes^a^	Data analysis
Chronic pain	1 screening item	✓	
Sociodemographic characteristics	Ad hoc questionnaire	✓	✓
Access to the study	Ad hoc question	✓	
IPV^b^ against women	European Union Agency for Fundamental Rights study on violence against women survey inventory		✓
Possible traumatic events experienced	Adapted version of Life Events Checklist for DSM-5	✓	✓
Disabling chronic pain	GCPS-R^c^		✓
Severity of PTSD^d^ symptoms	EGS-R^e^		✓
Gender norms and gender identity	CFNI-45^f^		✓
Activity patterns	APS^g^		✓
Exercising	IPAQ^h^		✓
Pain worrying (pain catastrophizing)	PCS^i^		✓
Pain-related self-efficacy	CPSS^j^		✓
Informal social support	ISSADI-PAIN^k^		✓
Health related quality of life	MOS SF-12^l^		✓
Physical functioning	BPI- SF^m^		
Anxiety and depression	HADS^n^		✓
Suicide attempts and thoughts	Ad hoc questionnaire		✓
Medication use and illegal drug use	Ad hoc questionnaire		✓
Health service use	Ad hoc questionnaire		✓

^a^We include compliance with the inclusion and exclusion criteria.

^b^IPV: intimate partner violence.

^c^GCPS-R: Graded Chronic Pain Scale Revised.

^d^PTSD: posttraumatic stress disorder.

^e^EGS-R: Escala de Gravedad de Síntomas Revisada (Posttraumatic Stress Disorder Symptom Severity Scale-Revised)

^f^CFNI-45: Conformity to Feminine Norms Inventory-45.

^g^APS: Activity Patterns Scale.

^h^IPAQ: International Physical Activity Questionnaire.

^i^PCS: Pain Catastrophizing Scale.

^j^CPSS: Chronic Pain Self-Efficacy Scale.

^k^ISSADI-PAIN: Informal Social Support for Autonomy and Dependence in Pain Inventory.

^l^MOS SF-12: Medical Outcomes Study Short-Form Health Survey-12.

^m^BPI-SF: Brief Pain Inventory–Short Form.

^n^HADS: Hospital Anxiety and Depression Scale.

#### Variables and Instruments for Control Purposes

### Screening for CP and Type and Sociodemographic Characteristics

These variables are described in detail in the phase 1 CP and Type and Sociodemographic Characteristics subsections.

### Access to the Study

Participants will have to indicate how they accessed the study (associations and support centers for IPV against women, CP associations, CP units, or primary health care centers).

#### Variables and Instruments for Risk and Protective Factors

### Sociodemographic Characteristics

Age, belonging to a minority group (yes or no), years of education, annual household and personal incomes (categorized as €0 to €19,000; €20,000 to €40,000; and >€40,000; 1€=US $1.14), relationship status (single, partnered, or married), relationship duration (years), relationship satisfaction (10-point scale), CP diagnosis (yes or no), and time since CP diagnosis (years).

### Gender Norms and Identity

Gender norms and identity will be measured using the Conformity to Feminine Norms Inventory-45 [135,136]. The Spanish-adapted version of the inventory [137] consists of 23 items rated on a 4-point response scale ranging from 0=*strongly disagree* to 3=*strongly agree*. The inventory includes 7 subscales: care for children, invest in appearance, domestic, romantic relationship, nice in relationships, thinness, and modesty. It is designed to assess the degree to which women conform to traditional femininity norms as reflected in their behaviors, feelings, and thoughts. Higher scores on this inventory indicate a greater adherence to these traditional femininity norms. The Conformity to Feminine Norms Inventory- has demonstrated good psychometric properties, making it a reliable measure for evaluating conformity to feminine norms.

### IPV Against Women: European Union Inquiry; Possible Traumatic Events Experienced: Adapted Version of Life Events Checklist for DSM-5; Disabling CP: Graded CP Scale Revised; Severity of PTSD Symptoms: Posttraumatic Stress Disorder Symptom Severity Scale-Revised

These scales have been described in study 1. With regard to the IPV against women questionnaire, participants will have to indicate whether they are currently experiencing such situations or have experienced them in the past and for how long. In addition, those women who have experienced IPV in the past will be asked how long they have lived free from violence.

#### Activity Patterns: Activity Patterns Scale

### Overview

The 3 activity patterns—pacing, avoidance, and persistence—can serve different goals and have varying impacts on health outcomes. To measure these patterns, we will use the Activity Patterns Scale [138]. This scale consists of 8 subscales, each with 3 items, for a total of 24 items. The subscales include pain avoidance, activity avoidance, task-contingent persistence, excessive persistence, pain-contingent persistence, pacing to increase activity levels, pacing to conserve energy for valued activities, and pacing to reduce pain. Participants will rate how well each statement applies to them on a 5-point scale ranging from 0=*not at all* to 4=*always*. Higher scores in a subscale indicate a stronger tendency to display the corresponding activity pattern. The instrument demonstrates adequate reliability as well as structural, convergent, and criteria validity. It has been specifically developed for use with the Spanish population [138].

### Exercising: International Physical Activity Questionnaire

Although some studies advocate the use of objective measures of physical activity and physical exercise [139], the International Physical Activity Questionnaire (IPAQ) [140] allows the collection of detailed information on physical activity performance comparable to that collected with objective measures such as pedometers [141]. The short, self-administered version of the IPAQ focuses on the past 7 days and consists of 7 items that estimate the time spent on moderate to vigorous physical activity, such as walking, bicycling, or heavy lifting, in at least 10-minute bouts over the week. This version provides a continuous score reflecting the minutes of physical activity performed, with higher scores indicating greater physical activity. The IPAQ presents good psychometric properties and is available in Spanish [140].

### Pain Worrying (Pain Catastrophizing): Pain Catastrophizing Scale

This scale [142] contains 13 items that are rated on a 5-point scale ranging from 0=*not at all* to 4=*all the time*. It assesses the 3 components of catastrophizing: rumination, magnification, and helplessness. The total score ranges from 0 to 52 points. Higher scores reflect higher levels of catastrophic thoughts. The Spanish version [143] presents strong psychometric properties.

### Pain-Related Self-Efficacy: CP Self-Efficacy Scale

This specific scale [144] for patients with CP helps measure perceived self-efficacy and capability to manage pain consequences. It is composed of 22 items structured across 3 factors: self-efficacy to control pain, self-efficacy for physical functioning, and self-efficacy for symptom control. It is a well-known instrument that has been validated with the Spanish population with good psychometric properties [145]. In the Spanish version items are rated from 0=*I think I am totally incapable* to 10=*I think I am totally capable*, with a higher score suggesting higher self-efficacy in a specific factor or in the overall measure of self-efficacy.

### Informal Social Support: Informal Social Support for Autonomy and Dependence in Pain Inventory

This instrument [146] consists of 11 items designed to measure both emotional and instrumental social support in relation to promoting functional autonomy or addressing functional dependence. Participants are asked to rate how frequently their family members have provided support when they were in pain. In our case we specifically ask on the support they receive from their intimate partners. Only women currently in a relationship will be required to complete this section. Responses are provided on a Likert scale ranging from 1=*not at all frequent* to 5=*extremely frequent*. Higher scores indicate that participants perceive more frequent support for functional autonomy or dependence. This instrument has demonstrated validity and reliability in its original form and has been validated with the Spanish population [146].

#### Variables and Instruments for Health Outcomes

### Health-Related Quality of Life: Short-Form Health Survey-12

This tool [147] consists of 12 items that measure both positive and negative health states across 8 health concepts (physical functioning, role limitations because of physical health problems, bodily pain, general health, vitality, social functioning, role limitations because of emotional problems, and mental health). It offers 2 physical and mental health summary measures. The responses are based on Likert scales ranging from 3 to 5 points. This questionnaire has been validated for use with the Spanish population [148].

### Physical Functioning: Brief Pain Inventory–Short Form

The Brief Pain Inventory–Short Form [149] assesses pain severity and pain interference. The latter includes 7 items that assess pain’s impact on various areas of life (general activity, mood, ability to walk, work, social relationships, sleep, and the ability to enjoy activities), which participants rate on a 10-point scale (maximum score=70). For the purpose of evaluating physical functioning, items related to general activity, ability to walk, and ability to work will be selected, with a maximum score of 30. This instrument has been validated for use with the Spanish population [150].

### Anxiety and Depression: Hospital Anxiety and Depression Scale

This scale [151] includes 2 subscales, one for anxiety and one for depression, each with 7 items and a 4-point response scale ranging from 0=*no presence or minimum presence* to 3=*maximum presence*. The scale assesses emotional distress in patients with chronic conditions, focusing on the cognitive and behavioral symptoms of anxiety and depression. The recommended cutoff scores for possible cases are 8 for both subscales, with scores of >10 indicating probable cases. This scale has been widely validated across multiple countries, including Spain, and demonstrates strong psychometric properties [152].

### Suicide Attempts and Thoughts: Ad Hoc Questionnaire

Participants will be asked whether they have ever considered committing suicide (yes or no); how frequently they have had such thoughts (never, rarely, sometimes, often, or always); and the number of suicide attempts, if any.

### Medication Use and Illegal Drug Use: Ad Hoc Questionnaire

Participants will be asked to indicate the types of analgesics prescribed for their CP condition and their adherence to the prescribed medication regimen. They will be asked if they are taking the medication as prescribed, not taking it at all, or taking it in lesser or greater amounts. The questionnaire will also inquire about the use of nonprescribed medications and the consumption of other legal (eg, alcohol) and illegal drugs (eg, marijuana) for pain management. In addition, participants will be asked about other prescribed medications they may be taking and the purposes for which these medications are used.

### Health Service Use: Ad Hoc Questionnaire

Participants will be asked about the frequency of visits to their general practitioner. Specifically, they will be asked how many times per month they visit their general practitioner, with the following response options: none, only when needing medication prescriptions or check-ups, at least once a month, or ≥2 times per month.

#### Data Analysis

Inferential statistics help researchers draw conclusions about populations based on sample data, providing insights into social phenomena [153]. Machine learning, as a subset of artificial intelligence (AI) techniques, analyzes large datasets to identify patterns and make predictions [154]. These methods are transformative in gender studies, uncovering patterns in gender-related data and deepening the understanding of social inequalities and biases [155]. These methods have been successfully applied to areas such as forecasting gender-based violence, further demonstrating their relevance in social sciences [156].

Exploratory data analysis (EDA) will be crucial in examining CP and its connection to past or current IPV against women. EDA uses techniques such as scatterplots, clustering algorithms, and principal component analysis to investigate multidimensional datasets [157]. Scatterplots help identify correlations between sociodemographic variables, clustering algorithms reveal subgroups within the population, and principal component analysis reduces dimensionality to uncover latent factors such as the relationship between gender identity and social support [158]. The use of EDA will allow us to detect groups and subgroups at a glance by creating subclusters, that is, more specific profiles in each of the groups.

Inferential statistical methods will be applied to differentiate between women with CP alone and those with CP who have experienced IPV in the past or are currently experiencing IPV. Hypothesis testing, estimation, and prediction techniques will be used to make comparisons, with visual tools such as boxplots for examining variable distributions across groups. We will use 2-tailed *t* tests and ANOVA to compare means across groups [159].

Feature ranking techniques will help identify the most relevant variables for accurate classification. Methods such as information gain, gain ratio, Gini index, and relief will determine the importance of features such as education, socioeconomic status, pain level, and social support [160]. This feature ranking will help prioritize the most influential factors affecting each group, including those based on country distribution.

Machine learning classifiers, including random forest, decision trees, adaptive boosting, gradient boosting, and support vector machines, will be applied to classify data. The process will involve training, validation, and testing phases, with hyperparameter tuning during validation. K-fold cross-validation will be used to evaluate the model’s performance, using training, validation, and testing phases across different folds [161]. The model’s performance will be assessed using metrics such as precision, recall, *F*_1_-score, and receiver operating characteristic curves [162].

Feature importance evaluation will identify which variables most significantly influence the model’s predictions. This analysis, combined with explanatory models such as Shapley additive explanations, will provide further insights into how features impact classification outcomes [163]. These methods are widely used in gender studies to uncover social patterns and inequalities. By combining Shapley additive explanations values with EDA, we aim to pinpoint the most significant factors that explain different profiles in the data [164].

Data analysis will be conducted using Python (Python Software Foundation), with libraries such as *pandas* (for data manipulation), *NumPy* (for numerical operations), *scikit-learn* (for machine learning), and the combination of *matplotlib* and *seaborn* (for visualization). This suite of tools provides an efficient framework for inferential statistical analysis and exploratory data. Comparable analysis could also be performed using R (R Foundation for Statistical Computing), which presents similar usability for data study. R comes with *tidyverse*—a package that merges *dplyr* and *ggplot2* for visualizing data—and *caret* for building predictive models. Similar software such as MATLAB (The MathWorks, Inc) also allows for wider functionality for statistical computing and algorithm development.

## Results

As of May 2025, we had contacted relevant associations to present the project and begin recruiting voluntary participants. We have conducted the pilot study to assure the adequacy of the questionnaire and usability or technical functionality of the web-based platform. The results are expected to be published starting in January 2026. The study timeline is presented in Table 3.

**Table 3:** Study timeline.

Year, study phases, and tasks				Months																						
				1		2		3		4		5		6		7		8		9		10		11		12
**Year 1**																										
	**Phase 1**																									
		Identification of and contact with associations and support centers for IPV^a^ against women, CP^b^ associations, CP units, and primary health care centers to present the projects and request their collaboration for phase 1 and phase 2	✓		✓																					
		Development of semistructured interview guide					✓																			
		Conducting interviews							✓		✓															
		Transferring data											✓													
		Data analysis													✓				✓							
		Writing articles and communications																	✓		✓		✓		✓	
**Year 2**																										
	**Phase 2**																									
		Contact with associations and support centers for IPV against women, CP associations, CP units, and primary health care centers to initiate participation in phase 2	✓		✓																					
		Design of web-based questionnaire	✓		✓																					
		Questionnaire piloting and inclusion of changes					✓																			
		Supervision of questionnaire completion							✓		✓		✓		✓				✓		✓		✓		✓	
**Year 3**																										
	Data analysis			✓		✓		✓																		
	Writing articles and communications									✓		✓		✓		✓				✓		✓		✓		✓
	Develop assessment and intervention guidelines																			✓		✓		✓		

^a^IPV: intimate partner violence.

^b^CP: chronic pain.

## Discussion

### Summary

This project has 2 main aims. The first is to acquire knowledge on the meaning and implications of living with CP for women experiencing IPV compared to those with CP alone. In this regard, we hypothesize that (1) women who experience any form of IPV (psychological, physical, or sexual) will report a greater daily impact of CP on their functionality than those with CP alone; (2) compared to the latter, the former will report a stronger bidirectional relationship between CP and intimate relationship dynamics as well as worse health outcomes (ie, episodes of violence will increase CP symptomatology and vice versa), with such a negative cycle being more prevalent; and (3) CP management and experiences with the health system will be better among women with CP alone. Hypotheses 1 and 3 are based on the fact that some authors have highlighted that the impact of IPV against women on the health of those affected adds to CP [31]. This could impair their ability to self-manage CP, leading to increased medication use and more frequent visits to health centers [165]. We believe that this result will be independent of the type of violence experienced because studies have shown that psychological violence alone can negatively impact CP severity and disability [166,167].

Regarding our second hypothesis, we expect that aggressors will present more hostility and criticism toward their partners’ health conditions as a way to undermine them, increase dependence, and control them, considering violence as an instrumental component [165]. We also expect that periods of violence will be linked to an increase in CP symptomatology. However, in women with CP alone, their partners will likely promote autonomy by encouraging and reinforcing healthy behaviors or promote dependence by maintaining disabling pain behaviors through solicitousness. In this group of women, even if hostile responses can also be present sometimes, their impact on CP will be less significant because they need to take place in a context of low marital satisfaction [95,96], and we assume that levels of marital satisfaction will be higher among women with CP alone. In addition, we expect that periods with an increase in CP symptomology will be more related to external stressors than to the relationship itself.

Second, we aim to identify whether women with CP alone, those with CP and currently experiencing IPV, and those with CP and a past history of IPV present different profiles based on risk and protective factors for (disabling) CP and health outcomes. We hypothesize that women with CP and current or past experiences of IPV will show higher levels of disabling pain, more excessive persistence or avoidance activity patterns, lower levels of exercise, worse pain-related self-efficacy, greater pain worrying, informal social support that fosters dependence, lower levels of physical functioning, greater anxiety and depression, poorer medication management, increased illegal drug use, more suicidal thoughts and attempts, and a higher frequency of health system use. This hypothesis is based on previous findings indicating higher levels of disabling pain in women experiencing IPV compared to those with CP alone, as well as higher levels of suicidal thoughts, addiction issues, and greater use of health services compared to the general female population [23,165]. These characteristics will likely worsen in the presence of PTSD [82]. In addition, because pain-related self-efficacy and pain worrying have been linked to CP [65-69,78], we expect these factors to most effectively account for the profiles of women with a current or past history of IPV, along with informal social support, for the aforementioned reasons.

Moreover, because AI techniques go beyond traditional inferential statistics, it can identify patterns and relationships between variables that might remain undetected by the human eye. AI may uncover specific profiles within different groups of women, revealing how factors such as educational background, traditional feminine roles, ethnicity, and the duration of violence intersect with CP-related variables; for example, it could be that the pattern of excessive persistence is more common in women who are in the initial stages of the relationship and that they may seek to conform to traditional gender roles in an effort to meet their aggressors’ expectations. AI’s ability to process large datasets could lead to new insights and advances in understanding pain, its management, and its relationship with other concurrent psychosocial factors.

### Limitations

This study is not without limitations. One of the main challenges is the high number of participants required for the quantitative study. To mitigate this issue, we will contact primary health care centers, associations and support centers for IPV against women, CP associations, and CP units across the province of Alicante. A second limitation is the use of nonprobability sampling, which may introduce bias. However, this research aims to provide a preliminary understanding of the co-occurrence of these 2 significant health issues; to the best of our knowledge, it will be the first to do so with a large sample using AI. A third limitation concerns the number of questionnaires that need to be completed in the second study. To address this, participants’ responses will be saved, and they will be given ample time to complete all questionnaires. In addition, the order of presentation will be randomized to reduce the likelihood that the same questionnaires are consistently left unanswered. We hope this will result in a similar number of responses across all questionnaires. To further encourage questionnaire completion, we will ask the center directors and professionals to remind participants about the importance of answering all questions.

A fourth limitation is the focus on only individual and relational factors. While community and societal factors also influence CP and its disabling effects [168], the primary aim of this study is to enhance the detection and treatment of CP in health care settings by personalizing care. Therefore, the focus is placed on individual and relational factors. Finally, not all scales have been validated with Spanish samples. Validating several scales has high costs and is time consuming, but we will conduct translation and back-translation procedures and test the instruments directly with the population. Moreover, the study will only be conducted in the province of Alicante. However, if profiles are identified, we plan to extend the second study to other Spanish regions as well as other countries while controlling for other macrovariables (eg, free access to the health system) to analyze the commonalities and differences in the profiles. If minimal or no differences are found, the profiles could be largely explained by the experience of IPV against women, and recommendations could be generalized to similar countries.

### Conclusions

In 2023, the European Pain Federation identified 7 key areas of focus in pain research [169], two of which are central to our study: (1) understanding and addressing comorbidities that complicate management and recovery; and (2) personalizing pain management because current treatments remain uniform, regardless of factors such as gender, age, lifestyle, or overall health. Our research directly addresses both areas. First, we tackle 2 closely related yet under-studied health issues: CP and IPV against women. As Craner et al [23] have noted, most research on traumatic events and CP has concentrated on childhood trauma and posttraumatic stress symptoms, with limited attention given to IPV against women. Second, our study aims to identify profiles based on risk factors and health outcomes, which could enhance detection and help design more personalized treatments.

In addition, our study could indirectly have a positive impact on costs and contribute to achieving the United Nations’ Sustainable Development Goals. Both CP and IPV against women have significant consequences for individuals and societies, with economic costs of at least €300 billion and €172 billion, respectively (US $343 billion and US $197 billion) [4,18]. These high costs primarily stem from frequent primary health care consultations, diagnostic procedures, medication, therapies (eg, psychology, occupational therapy, infiltrations, or surgery), hospitalizations, and loss of work productivity. As a result, both health issues severely affect productivity and the health care system [121,170], threatening human capital and hindering progress toward the United Nations’ Sustainable Development Goals. We believe that by improving the detection of IPV against women associated with CP and tailoring treatments to the identified risk factors and health outcomes, our study can help reduce costs and advance progress toward achieving the United Nations’ Sustainable Development Goals. Effective self-management could alleviate the burden that CP places on the health care system and reduce its economic costs [171,172].

## Abbreviations

AI: artificial intelligence
CP: chronic pain
EDA: exploratory data analysis
IPAQ: International Physical Activity Questionnaire
IPV: intimate partner violence
PTSD: posttraumatic stress disorder

## Data Availability

Data sharing is not applicable to this paper as no datasets were generated or analyzed during this study. However, when generated, the datasets as well as the interview guide (phase 1) will be available from the corresponding author on reasonable request.
